# Effects of combining exercise with a 16:8 time-restricted eating protocol on body composition and glucose–lipid metabolism in adults: a systematic review and meta-analysis

**DOI:** 10.3389/fnut.2026.1816555

**Published:** 2026-05-14

**Authors:** Hengru Liu, Guidan Hu, Xiujie Ma

**Affiliations:** 1 School of Wushu, Chengdu Sport University, Chengdu, China; 2 School of Journalism, Media and Culture, Cardiff University, Cardiff, United Kingdom

**Keywords:** body composition, cardiometabolic risk, exercise training, intermittent fasting, lipid metabolism, meta-analysis, obesity, time-restricted eating

## Abstract

**Background:**

Time-restricted eating (TRE) has emerged as a promising nutritional strategy for improving metabolic health, yet its additive benefits when combined with structured exercise remain unclear.

**Objective:**

This systematic review and meta-analysis evaluated the effects of exercise combined with a 16:8 TRE protocol on body composition and glucose–lipid metabolism in adults.

**Methods:**

Following PRISMA guidelines and PROSPERO registration (CRD420251240058), eight electronic databases were searched through August 2025 for randomized and randomized crossover trials comparing exercise plus 16:8 TRE with exercise alone. Three reviewers independently performed study selection, data extraction, risk-of-bias assessment (ROB 2), and GRADE evaluation. Random-effects meta-analyses were conducted using R software. Subgroup analyses and meta-regression explored potential moderators.

**Results:**

Fifteen trials involving 511 participants were included. Compared with exercise alone, the combined intervention significantly reduced body weight (MD − 1.44 kg, 95% CI − 2.74 to −0.13) and fat mass (MD − 1.04 kg, 95% CI − 1.95 to −0.13). Significant improvements were also observed in triglycerides (SMD − 0.25, 95% CI − 0.45 to −0.06) and LDL cholesterol (SMD − 0.22, 95% CI − 0.37 to −0.08), while other body composition and glycemic markers showed no significant changes. Greater reductions in body weight and fat mass were observed when TRE was combined with aerobic exercise, and LDL-C reductions were more pronounced in men. Sensitivity analyses confirmed result robustness with minimal publication bias.

**Conclusion:**

Exercise combined with a 16:8 TRE protocol may confer small additional short-term benefits for body weight, fat mass, and selected lipid markers beyond exercise alone. However, the evidence base is limited by the small number of studies, modest sample sizes, and imbalance in participant characteristics across sex and metabolic status. These findings should therefore be interpreted cautiously.

## 1 Introduction

Obesity and its associated metabolic disorders remain major public health challenges in the 21st century. In this review, “body composition” refers to total body weight, fat mass, body fat percentage, fat-free mass/lean body mass, and waist circumference as an indicator of central adiposity. Excess adiposity is clinically important because it increases the risk of insulin resistance, type 2 diabetes, cardiovascular disease, metabolic syndrome, and non-alcoholic fatty liver disease (1–4). Importantly, BMI alone does not fully capture adiposity burden or fat distribution. Central fat accumulation, especially abdominal or visceral adiposity, is associated with cardiometabolic risk independent of BMI, and adults with a normal BMI may still present with abdominal obesity and unfavorable metabolic profiles (5, 6).

Time-restricted eating (TRE), a form of intermittent fasting, intentionally limits daily food and energy intake to a consistent eating window without necessarily prescribing caloric restriction (7, 8). Among TRE models, the 16:8 protocol—consisting of a 16-h fasting window followed by an 8-h eating window—is one of the most widely adopted because it is operationally simple and often feasible in free-living settings (48). Current evidence suggests that meal-timing interventions may produce small reductions in body weight when sustained for at least 12 weeks, although certainty is constrained by heterogeneity and risk of bias (8). Proposed mechanisms include better alignment of feeding–fasting cycles with circadian biology, altered substrate oxidation, and downstream effects on glucose and lipid handling (49–51), but the magnitude of these effects appears to depend on intervention duration, eating-window timing (52, 53), baseline metabolic status, and concurrent changes in total energy intake (8–10, 54, 55).

Exercise, by contrast, has well-established independent effects on adiposity and cardiometabolic health. Aerobic exercise shows a dose–response association with reductions in body weight, waist circumference, and body fat (56), while resistance training supports lean-mass preservation and improves glycemic and lipid regulation (11, 12). Moreover, broader diet-plus-exercise meta-analyses indicate that combining dietary modification with exercise can improve triglycerides and LDL-C more than exercise alone, suggesting that behavioral integration may yield additive benefits (13). However, the incremental value of meal timing per se remains unclear because much of the existing diet–exercise literature centers on caloric restriction rather than TRE.

Related work on TRE combined with caloric restriction has shown significant reductions in body weight, fat mass, and waist circumference, but less consistent improvement in glucose–lipid markers (14, 57, 58). This distinction is important because TRE is conceptually different from traditional energy restriction: it manipulates the timing of intake, whereas caloric restriction directly prescribes the amount of intake. Accordingly, the novelty of the present review lies in isolating whether a 16:8 TRE schedule confers additional benefits when superimposed on structured exercise and compared with an exercise-matched control without TRE.

Nonetheless, findings from clinical trials on the combined effects of TRE and exercise remain inconsistent. Some studies have reported significant reductions in fat mass or improvements in metabolic markers, whereas others have found little added benefit beyond exercise alone, particularly over short interventions or in metabolically healthy samples. In this review, “moderating factors” refers to characteristics that may influence intervention effects, including exercise modality, sex, intervention duration, baseline BMI or metabolic status, baseline energy intake, and, where reported, timing of exercise or the eating window. Therefore, the present study aimed to synthesize and quantitatively evaluate the current evidence on the effects of combining exercise with 16:8 TRE on body composition and glucose–lipid metabolism in adults, and to explore which study or participant characteristics may modify these effects.

## 2 Materials and methods

This study followed the PRISMA guidelines for systematic reviews and meta-analyses (15) to ensure transparency of the research process and methodological rigor, and was prospectively registered on the PROSPERO platform under registration number CRD420251240058.

### 2.1 Literature search

A comprehensive literature search was conducted between August 10 and August 15, 2025 across the following databases: PubMed, Cochrane Library, Embase, Scopus, Web of Science Core Collection, China National Knowledge Infrastructure (CNKI), Wanfang Data, and VIP Database. The search covered database inception through August 1, 2025. Because indexing status changes over time, searches replicated after this cutoff may yield larger numbers of records than the dataset screened for the present review.

The Chinese search strategy used combinations of terms such as:

(“限时进食” OR “限时饮食” OR “限时进餐” OR “间歇性禁食” OR “热量限制”) AND (“运动” OR “训练” OR “体力活动” OR “锻炼” OR “有氧运动” OR “抗阻训练”).

The English search strategy was as follows:

(“time-restricted eating” OR “time-restricted diet” OR “time-restricted feeding” OR “intermittent fasting” OR “caloric restriction”) AND (“exercise*” OR “training” OR “activit*” OR “motion” OR “aerobic exercise” OR “resistance training”).

Additionally, backward citation searching of reference lists from included studies and relevant reviews, as well as forward citation tracking in citation databases, was undertaken to maximize literature coverage. No search update was performed after the August 2025 search closure.

### 2.2 Study selection

All references were imported into EndNote 21 for deduplication. Title and abstract screening were independently conducted by the first author according to predefined inclusion and exclusion criteria. Discrepancies were resolved through discussion with the second author. If consensus could not be reached, the corresponding author made the final decision. Full-text articles were then independently reviewed by both the first and second authors. Any disagreements were handled using the same decision-making process applied during the title and abstract screening phase.

### 2.3 Inclusion and exclusion criteria

Inclusion criteria:

1. Population: Adults aged ≥18 years, regardless of sex, obesity status, or baseline health condition.
2. Intervention: The control group received structured exercise alone, including aerobic training, resistance training, or a combination of both, together with a habitual diet or an unrestricted eating window without a TRE prescription. The experimental group received the same exercise program plus a 16:8 time-restricted eating intervention, strictly defined as 16 h of fasting followed by an 8-h eating window. Studies were eligible whether or not total energy intake was matched, provided caloric intake and the exercise protocol were sufficiently described.
3. Comparison: Studies comparing exercise alone with the combined exercise + TRE intervention.
4. Outcomes: Changes in body composition indicators (body weight, BMI, body fat percentage, fat mass, fat-free mass, and waist circumference as a proxy of central adiposity) and/or glucose–lipid metabolism markers (e.g., triglycerides, total cholesterol, HDL-C, LDL-C, fasting glucose, fasting insulin, HOMA-IR).
5. Study design: Randomized controlled trials (RCTs) or randomized crossover trials, with a minimum washout period of 2 weeks for the latter.

Exclusion criteria:

1. Studies that incorporated other confounding interventions likely to obscure the independent effects of TRE or exercise (e.g., anti-obesity or hypoglycemic medications, bariatric surgery, or multimodal programs in which the specific contribution of TRE plus exercise could not be isolated).
2. Animal studies, review articles, case reports, conference abstracts, study protocols, or editorials.
3. Studies with inaccessible full texts, incomplete data, or data that could not be extracted or converted.
4. For mixed samples (e.g., overweight and obesity combined), studies were retained if the combined sample met the other eligibility criteria; classification was based on the study authors’ description, and baseline BMI was extracted to aid interpretation.

### 2.4 Data extraction

Data extraction was independently performed by the first and third authors using a standardized extraction form created in Excel. For studies with missing data or results presented only in graphical form, the authors of the original studies were contacted to request complete datasets. If no response was received, GetData Graph Digitizer 2.26 was used to extract numerical values from figures. Extracted data included study characteristics, participant characteristics, exercise modality, exercise frequency and session duration when reported, comparator diet description, TRE window timing when available, intervention duration, and means and standard deviations (SD) for each outcome. If only 95% confidence intervals (CIs) or standard errors (SEs) were reported, SDs were calculated according to formulas provided in the Cochrane Handbook (16). Studies with insufficient data after these efforts were excluded from the meta-analysis.

### 2.5 Risk of bias assessment

Risk of bias was independently assessed by the first and third authors using the Cochrane Risk of Bias 2.0 (ROB 2) tool (17). Disagreements were resolved through discussion; if consensus could not be achieved, a third reviewer was consulted to make the final judgment.

### 2.6 Quality of evidence assessment

The quality of evidence for each outcome was independently assessed by the first and corresponding authors using the GRADE (Grading of Recommendations Assessment, Development and Evaluation) approach (18).

### 2.7 Statistical analysis

For outcomes with consistent measurement units (e.g., body composition indicators), mean difference (MD) was used to calculate pooled effect sizes. For metabolic outcomes reported using varying units (e.g., mg/dL vs. mmol/L), standardized mean difference (SMD) with Hedges’ g correction was applied (19). All statistical analyses and visualizations were conducted using R software (version 4.4.2), primarily with the metafor and ggplot2 packages. Pooled effect sizes and their 95% confidence intervals were estimated using the restricted maximum likelihood (REML) method. Maximum likelihood (ML) estimation was applied for model comparison to more accurately account for between-study heterogeneity (20). Heterogeneity was primarily assessed using the *I*^2^ statistic, with values <25% considered low heterogeneity. Additional indices such as *τ*^2^ were also reported (21). For outcomes with significant pooled effects (*p* < 0.05), subgroup analyses (based on categorical variables such as sex and intervention type) and meta-regression (for continuous moderators such as age or BMI) were conducted to explore potential sources of heterogeneity (22). Both linear and non-linear regression models were considered to optimize interpretability and applicability (23). Sensitivity analysis was conducted by sequentially omitting individual studies to examine the robustness of the results. Publication bias was assessed using Egger’s regression test, with a *p*-value > 0.05 indicating no significant publication bias (24).

## 3 Results

### 3.1 Literature search and characteristics of included studies

A total of 2,063 records were identified through database searches completed in August 2025. After removing duplicates, 687 records remained. Based on predefined inclusion and exclusion criteria, 12 studies were retained after screening titles, abstracts, and full texts. An additional three studies were identified through snowballing methods (e.g., citation tracking and website searches).

**Figure 1:**
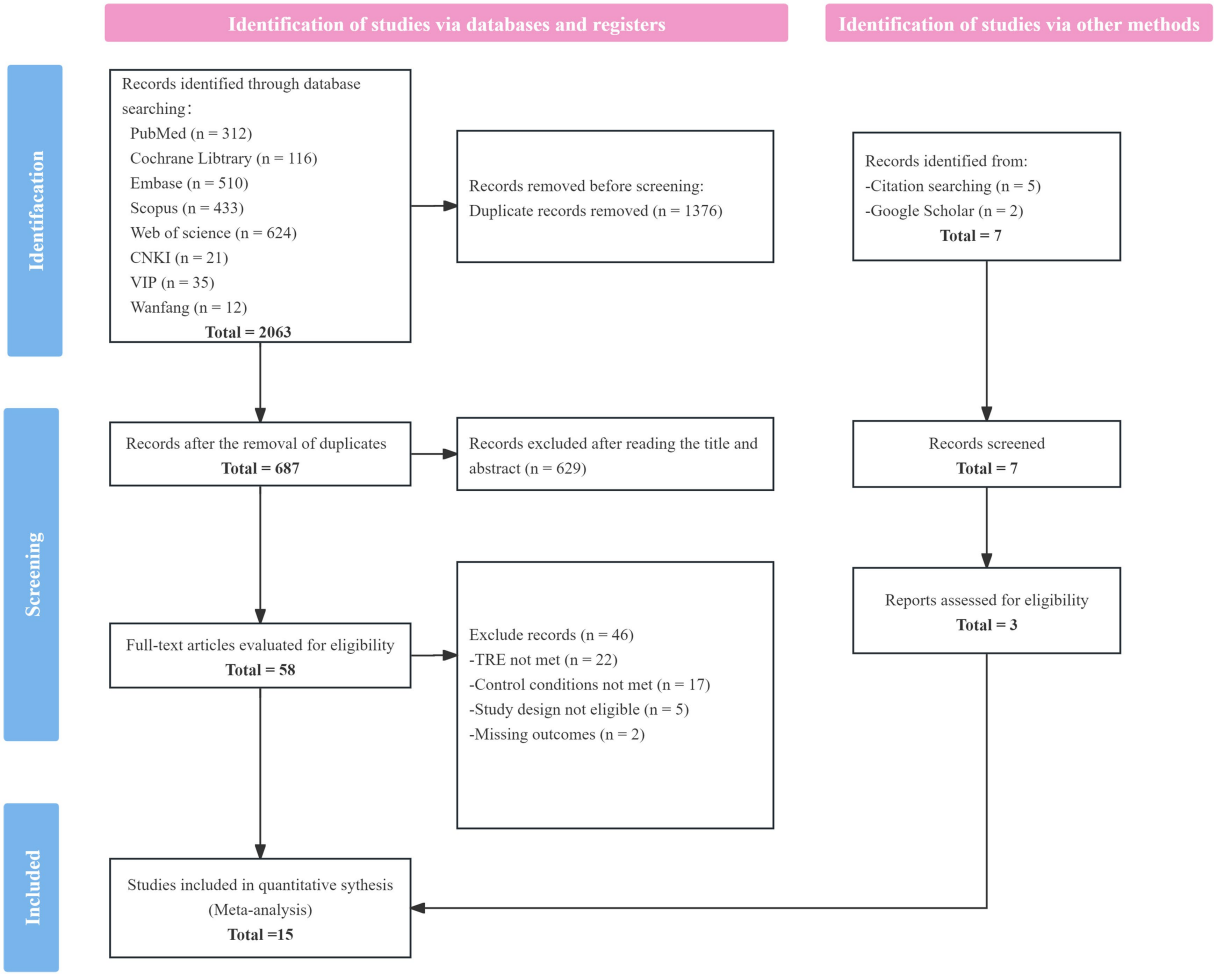
Flowchart of literature search.

In total, 15 studies were included in the final meta-analysis. The PRISMA flow diagram is presented in Figure 1.

Table 1 summarizes the characteristics of the included studies. A total of 511 participants were involved. Participant ages ranged from 19.38 to 54.20 years, with baseline BMI values between 18.50 and 32.50 kg/m^2^. Studies included both single-sex and mixed-sex cohorts, as well as healthy and overweight/obese samples. Aerobic protocols were most common, followed by resistance training and combined aerobic plus resistance training. In Table 1, the comparator labeled “normal diet” refers to a habitual diet or an unrestricted eating window without an explicit TRE prescription. Intervention durations ranged from 4 to 8 weeks. Where original studies reported BMI eligibility ranges rather than sample means, the published format was retained and clarified in the table note.

**Table 1:** Basic characteristics of included literature.

Study	Country/region	Study design	Age (years)	BMI (kg/m^2^)	Population	Sample size (N)	Intervention	Intervention duration (weeks)	Outcome measures
Correia et al. (38)	Portugal	RCT (crossover)	C: 23.7 ± 2.6	18.5–24.9	Healthy men	15	Normal diet + aerobic exercise	4	①②③④⑥⑦⑩⑨⑧
			T: 23.7 ± 2.6	18.5–24.9	Healthy men	15	Time-restricted eating + aerobic exercise	4	
Richardson et al. (39)	United States	RCT (crossover)	C: 28.7 ± 5.2	23.3 ± 3.2	Healthy men	15	Normal diet + aerobic exercise	4	①②③④⑥⑦⑩⑨⑧⑪⑫
			T: 28.7 ± 5.2	23.3 ± 3.2	Healthy men	15	Time-restricted eating + aerobic exercise	4	
Correia et al. (26)	Portugal	RCT (crossover)	C: 23.7 ± 2.6	/	Healthy men	18	Normal diet + resistance training	4	①②④
			T: 23.7 ± 2.6	/	Healthy men	18	Time-restricted eating + resistance training	4	
Liu et al. (40)	China	RCT (parallel)	C: 20.09 ± 1.35	21.35 ± 1.56	Healthy women	20	Normal diet + aerobic exercise	8	①③⑥⑦⑨⑧
			T: 19.93 ± 0.61	21.86 ± 1.48	Healthy women	19	Time-restricted eating + aerobic exercise	8	
Haganes et al. (41)	Norway	RCT (parallel)	C: 34.9 ± 7.0	32.5 ± 4.5	Women with obesity	33	Normal diet + aerobic exercise	7	①②⑥⑦⑩⑨⑧⑪⑫
			T: 37.3 ± 5.7	31.4 ± 4.0	Women with obesity	32	Time-restricted eating + aerobic exercise	7	
Lin et al. (42)	China	RCT (parallel)	C: 54.2 ± 7.9	25.7 ± 3.8	Women with obesity	33	Normal diet + aerobic exercise	8	①③④⑤⑥⑦⑩⑨⑧⑪⑫
			T: 50.1 ± 7.5	25.9 ± 3.7	Women with obesity	30	Time-restricted eating + aerobic exercise	8	
Moro et al. (43)	Italy	RCT (parallel)	C: /	/	Healthy men	10	Normal diet + resistance training	8	①②④⑥⑦⑩⑨⑧⑪⑫
			T: /	/	Healthy men	10	Time-restricted eating + resistance training	8	
Isenmann et al. (44)	Germany	RCT (parallel)	C: 27.4 ± 5.8	25.7 ± 3.3	Adults with obesity	17	Normal diet + combined aerobic and resistance training	8	①②⑤
			T: 27.9 ± 5.3	26.3 ± 3.0	Adults with obesity	18	Time-restricted eating + combined aerobic and resistance training	8	
Tovar et al. (45)	United States	RCT (crossover)	C: 28.7 ± 5.2	23.28 ± 3.23	Healthy men	27	Normal diet + aerobic exercise	4	①②③⑩
			T: 28.7 ± 5.2	23.28 ± 3.23	Healthy men	27	Time-restricted eating + aerobic exercise	4	
Kotarsky et al. (46)	United States	RCT (parallel)	C: 44 ± 2	29.8 ± 0.8	Adults with obesity	10	Normal diet + combined aerobic and resistance training	8	①②⑤⑦⑧⑪
			T: 45 ± 3	29.4 ± 0.8	Adults with obesity	11	Time-restricted eating + combined aerobic and resistance training	8	
Correia et al. (47)	Portugal	RCT (crossover)	C: 22.4 ± 2.8	24.2 ± 2.0	Healthy men	12	Normal diet + resistance training	4	①②③④
			T: 22.4 ± 2.8	24.2 ± 2.0	Healthy men	12	Time-restricted eating + resistance training	4	
Stratton et al. (25)	United States	RCT (parallel)	C: 22.5 ± 2.2	26.43 ± 3.12	Healthy men	13	Normal diet + resistance training	4	①②③
			T: 22.9 ± 3.6	25.85 ± 2.37	Healthy men	13	Time-restricted eating + resistance training	4	
Moro et al. (27)	Italy	RCT (parallel)	C: 19.38 ± 1.60	22.47 ± 1.83	Healthy men	8	Normal diet + aerobic exercise	4	①③④⑥⑦⑩⑪
			T: 19.38 ± 2.39	21.85 ± 1.65	Healthy men	8	Time-restricted eating + aerobic exercise	4	
Tinsley et al. (29)	United States	RCT (parallel)	C: 22.0 ± 2.4	24.3 ± 3.9	Healthy men	8	Normal diet + resistance training	8	②③
			T: 22.9 ± 4.1	27.2 ± 5.4	Healthy men	10	Time-restricted eating + resistance training	8	
Moro et al. (28)	Italy	RCT (parallel)	C: 28.47 ± 3.48	27.2 ± 4.3	Healthy men	17	Normal diet + resistance training	8	①②④⑥⑦⑩⑨⑧⑪
			T: 29.94 ± 4.07	26.5 ± 4.2	Healthy men	17	Time-restricted eating + resistance training	8	

C denotes the control group and T denotes the intervention group. “Normal diet” indicates a habitual diet or an unrestricted eating window without a TRE prescription. BMI values are reported as mean ± SD when available; where the original article reported eligibility ranges or category bands only, those original values are retained. For studies enrolling samples around the overweight/obesity threshold, participant descriptors follow the source article and should be interpreted alongside baseline BMI. RCT (parallel) denotes randomized controlled trials, and RCT (crossover) denotes randomized crossover trials.

① body weight; ② body fat mass; ③ body fat percentage; ④ fat-free mass; ⑤ waist circumference; ⑥ triglycerides; ⑦ total cholesterol; ⑧ high-density lipoprotein cholesterol; ⑨ low-density lipoprotein cholesterol; ⑩ fasting blood glucose; ⑪ fasting insulin; ⑫ insulin resistance.

### 3.2 Risk of bias assessment

**Figure 2:**
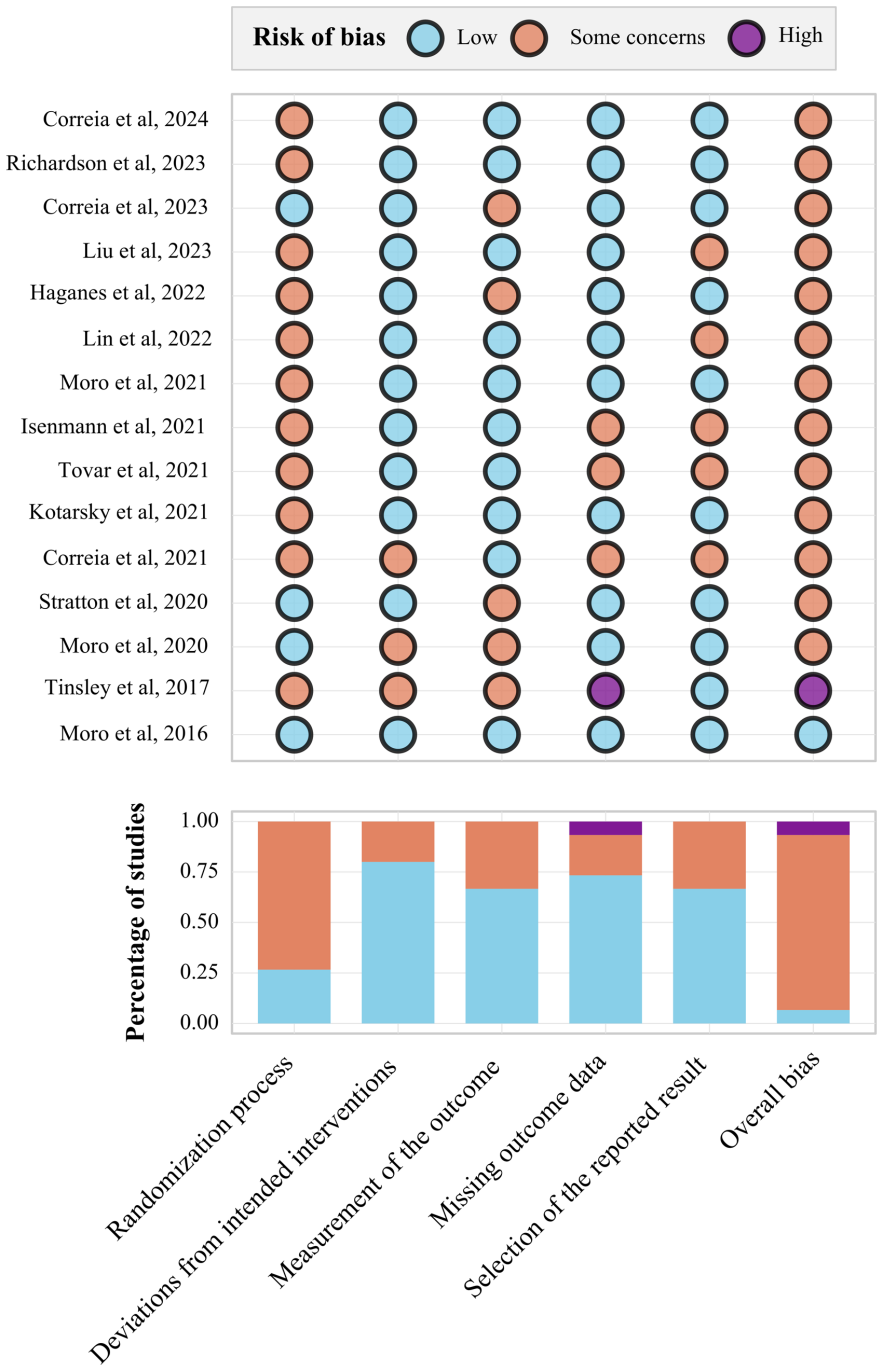
Risk of bias results graph. In the figure, the study name is represented by the author and year, followed by a reference number in brackets [e.g., (37)] indicating the corresponding citation. Risk of bias refers to the methodological quality assessment. Low: Low risk of bias. Some concerns: Some concerns regarding bias. High: High risk of bias.

Using the ROB 2.0 tool, low risk for the randomization process was identified in four studies (25–28), whereas the remaining studies raised some concerns, largely because allocation concealment or sequence-generation procedures were insufficiently reported. Low risk for deviations from intended interventions was observed in 11 studies, low risk for measurement of the outcome in 10 studies, and low risk for selection of the reported result in 10 studies. Missing outcome data was judged low risk in 11 studies; one study (29) was rated high risk in this domain. Overall, only Moro et al. (28) was judged low risk, Tinsley et al. (29) was judged high risk, and the remaining 13 studies were rated as having some concerns. These patterns indicate generally acceptable intervention delivery and outcome assessment, but limited reporting transparency for randomization and selective reporting (Figure 2).

### 3.3 Meta-analysis results

#### 3.3.1 Effects on body composition

**Figure 3:**
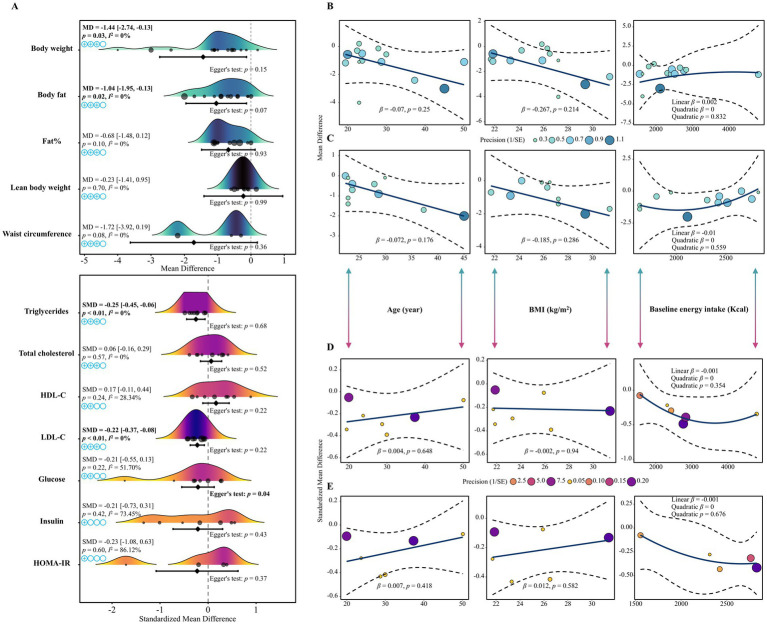
Summary of the main meta-analysis results and meta-regression analysis for the effects of exercise combined with 16:8 time-restricted eating on body composition and glucose–lipid metabolism. Panel **(A)** shows the pooled effect sizes for each primary outcome; panels **(B–E)** show the results of the meta-regression analyses for body weight, fat mass, triglycerides, and low-density lipoprotein cholesterol, respectively. The quality of evidence for each outcome was assessed using the GRADE system: ⊕◯◯◯ = very low quality; ⊕⊕◯◯ = low quality; ⊕⊕⊕◯ = moderate quality; ⊕⊕⊕⊕ = high quality.

Pooled estimates from 15 studies showed that exercise combined with 16:8 TRE significantly reduced body weight (MD = −1.44, 95% CI: −2.74 to −0.13, *p* = 0.03, *I*^2^ = 0%, moderate certainty) and fat mass (MD = −1.04, 95% CI: −1.95 to −0.13, *p* = 0.02, *I*^2^ = 0%, moderate certainty), compared with exercise alone, representing modest but directionally consistent incremental benefits. No significant differences were observed in body fat percentage, fat-free mass, or waist circumference (*p* > 0.05). These findings are broadly consistent with recent meta-analyses of TRE plus exercise, although the present review focused specifically on the 16:8 protocol and exercise-matched controls (30, 31). Sensitivity analyses confirmed the robustness of these findings with no significant heterogeneity across studies (*p* > 0.05). See Figure 3A.

Figures 3B,C and Table 2 present the results of subgroup and meta-regression analyses for body weight and fat mass. The combination of aerobic exercise and 16:8 TRE demonstrated significantly greater reductions in: Body weight (MD = −2.81, 95% CI: −5.29 to −0.33, *p* < 0.01, *I*^2^ = 0%, *τ*^2^ = 0); Fat mass (MD = −1.78, 95% CI: −3.36 to −0.19, *p* = 0.03, *I*^2^ = 0%, *τ*^2^ = 0). No significant moderating effects were observed for age, BMI, baseline energy intake, sex, intervention duration, or study design (all *p* > 0.05).

**Table 2:** Subgroup analysis results for significant body composition outcomes of exercise combined with 16:8 time-restricted eating.

Subgroup	Body weight								Fat mass							
	K (N)	MD	*p_d_*	95%LCI	95%UCI	*p_m_*	*I^2^*	*tau^2^*	K (N)	MD	*p_d_*	95%LCI	95%UCI	*p_m_*	*I^2^*	*tau^2^*
Gender						0.44	0.00%	0						0.49	0.00%	0
Male	11 (155)	−0.85	0.41	−2.85	1.16				10 (147)	−0.63	0.28	−1.77	0.51			
Female	3 (29)	−1.01	0.40	−3.39	1.36				1 (32)	−1.70	0.49	−6.54	3.14			
Mixed	2 (29)	−2.81	0.03	−5.29	−0.33				2 (29)	−1.78	0.03	−3.36	−0.19			
Intervention duration (weeks)						0.53	0.00%	0						0.65	0.00%	0
4	8 (118)	−0.82	0.48	−3.09	1.46				7 (110)	−0.61	0.35	−1.90	0.67			
7	1 (32)	−2.40	0.42	−8.24	3.44				1 (32)	−1.70	0.49	−6.54	3.14			
8	7 (115)	−1.69	0.05	−3.34	−0.04				5 (66)	−1.46	0.03	−2.80	−0.12			
Exercise type						<0.01	0.00%	0						0.03	0.00%	0
Aerobic exercise	7 (146)	−2.81	0.03	−5.29	−0.33				4 (89)	−1.78	0.03	−3.36	−0.19			
Resistance training	7 (90)	−0.63	0.65	−3.35	2.09				7 (90)	−0.44	0.57	−1.97	1.08			
Combined aerobic and resistance training	2 (29)	−1.05	0.27	−2.90	0.80				2 (29)	−0.96	0.03	−1.83	−0.09			
Study design						0.58	0.00%	0						0.39	0.00%	0
RCT (parallel)	11 (178)	−1.65	0.03	−3.17	−0.14				8 (121)	−1.41	0.03	−2.65	−0.17			
RCT (crossover)	5 (87)	−0.81	0.53	−3.37	1.75				5 (87)	−0.61	0.37	−1.95	0.73			

K = number of studies; N = sample size. p_d denotes the *p* value for the test of the overall effect; p_m denotes the *p* value for the test of between-subgroup differences; LCI = lower confidence limit; UCI = upper confidence limit.

#### 3.3.2 Effects on glucose–lipid metabolism

Pooled analyses revealed that exercise combined with 16:8 TRE significantly reduced: Triglycerides (SMD = −0.25, 95% CI: −0.45 to −0.06, *p* < 0.01, *I*^2^ = 0%, moderate certainty); LDL-C (SMD = −0.22, 95% CI: −0.37 to −0.08, *p* < 0.01, *I*^2^ = 0%, moderate certainty). However, no significant changes were observed for total cholesterol, HDL-C, fasting glucose, fasting insulin, or HOMA-IR (*p* > 0.05). These effects were modest in magnitude and should be interpreted as incremental improvements beyond exercise alone rather than stand-alone therapeutic effects. Sensitivity analyses confirmed the stability of these pooled results, with no significant between-study heterogeneity (*p* > 0.05). See Figure 3A.

Figures 3D,E and Table 3 display subgroup and meta-regression analyses for triglycerides and LDL-C. The results indicated that in male participants, the combined intervention led to significantly greater reductions in LDL-C: LDL-C (male subgroup): SMD = −0.36, 95% CI: −0.58 to −0.14, *p* < 0.01, I^2^ = 0%, *τ*^2^ = 0; between-subgroup difference by sex: p_m = 0.02. No significant moderating effects were found for age, BMI, baseline energy intake, exercise type, intervention duration, or study design (*p* > 0.05).

**Table 3:** Subgroup analysis results for significant glucose–lipid metabolism outcomes of exercise combined with 16:8 time-restricted eating.

Subgroup	Triglycerides								Low-density lipoprotein cholesterol							
	K (N)	SMD	*p_d_*	95%LCI	95%UCI	*p_m_*	*I^2^*	*tau^2^*	K (N)	SMD	*p_d_*	95%LCI	95%UCI	*p_m_*	*I^2^*	*tau^2^*
Gender						0.24	0.00%	0						0.02	0.00%	0
Male	5 (65)	−0.36	0.01	−0.62	−0.10				4 (57)	−0.36	0.00	−0.58	−0.14			
Female	3 (81)	−0.13	0.36	−0.41	0.15				3 (81)	−0.11	0.30	−0.31	0.10			
Intervention duration (weeks)						0.97	0.00%	0						0.56	0.00%	0
4	3 (38)	−0.29	0.12	−0.64	0.07				2 (30)	−0.37	0.03	−0.69	−0.04			
7	1 (32)	−0.23	0.31	−0.68	0.22				1 (32)	−0.13	0.37	−0.42	0.16			
8	4 (76)	−0.24	0.07	−0.51	0.02				4 (76)	−0.21	0.04	−0.42	−0.01			
Exercise type						0.26	0.00%	0						0.29	0.00%	0
Aerobic exercise	6 (119)	−0.44	0.02	−0.83	−0.06				5 (111)	−0.18	0.04	−0.35	−0.01			
Resistance training	2 (27)	−0.19	0.09	−0.41	0.03				2 (27)	−0.36	0.02	−0.66	−0.06			
Study design						0.97	0.00%	0						0.33	0.00%	0
RCT (parallel)	6 (116)	−0.25	0.02	−0.47	−0.04				5 (108)	−0.19	0.03	−0.35	−0.02			
RCT (crossover)	2 (30)	−0.26	0.24	−0.69	0.17				2 (30)	−0.37	0.03	−0.69	−0.04			

K = number of studies; N = sample size. p_d denotes the *p* value for the test of the overall effect; p_m denotes the *p* value for the test of between-subgroup differences; LCI = lower confidence limit; UCI = upper confidence limit.

### 3.4 Publication bias

Egger’s regression tests, presented in Figure 3, indicated no significant publication bias for most outcomes (*p* > 0.05), except for fasting glucose (*p* = 0.04), where potential publication bias was detected.

### 3.5 GRADE quality assessment

The GRADE assessment indicated moderate-certainty evidence for body weight, fat mass, triglycerides, and LDL-C, primarily downgraded once for study limitations related to reporting and randomization concerns. Evidence for body fat percentage, fat-free mass, waist circumference, total cholesterol, HDL-C, and fasting insulin was judged low certainty because of imprecision and, for some outcomes, inconsistency. Evidence for fasting glucose and HOMA-IR was considered very low because of substantial heterogeneity and imprecision. Accordingly, confidence is greatest for the modest effects observed on body weight, fat mass, triglycerides, and LDL-C, and substantially lower for the remaining outcomes.

## 4 Discussion

This meta-analysis quantitatively synthesized data from 15 studies to evaluate the effects of combining exercise with a 16:8 TRE protocol on body composition and glucose–lipid metabolism in adults, while also exploring potential moderating factors. Compared with exercise alone, adding a 16:8 TRE schedule produced modest incremental reductions in body weight, fat mass, triglycerides, and LDL-C, with larger body-composition effects observed in studies using aerobic exercise. This pattern suggests that the combined intervention may augment, rather than replace, the established benefits of exercise. The findings should therefore be interpreted against three relevant comparator literatures: exercise-only interventions, TRE without exercise, and TRE combined with caloric restriction.

### 4.1 Effects of exercise combined with 16:8 time-restricted eating on body composition

The present study demonstrated that, relative to exercise alone, combining exercise with a 16:8 TRE regimen yielded an additional mean reduction of 1.44 kg in body weight and 1.04 kg in fat mass. From a clinical perspective, these effect sizes are modest and unlikely, on their own, to constitute large therapeutic change over 4–8 weeks; however, they may still be meaningful for individuals pursuing gradual fat loss while maintaining structured exercise. This pattern aligns with the systematic review and meta-analysis by Dai et al. (30) and with Hays et al. (31), who reported reductions in fat mass with TRE in exercising adults. At the same time, the magnitude is smaller than that reported in broader exercise or diet-plus-exercise literatures, where exercise dose and/or caloric restriction are often more intensive or longer in duration (12, 14).

Mechanistically, the additional fat-loss signal may reflect alignment of feeding–fasting cycles with circadian metabolism, longer fasting-related reliance on lipid oxidation, and the preservation of exercise-stimulated energy expenditure. However, these explanations remain provisional because most included trials did not report the timing of exercise relative to the eating window, nor did they systematically examine early versus late TRE schedules. This omission is important because timed exercise—especially morning exercise—can shift circadian phase and may interact with meal timing and substrate use (32, 33). Future trials should explicitly report whether exercise occurred in the fasting or feeding period and whether exercise was performed in the morning, afternoon, or evening.

Our subgroup analysis suggests that aerobic exercise may show a more favorable synergy with 16:8 TRE for short-term reductions in body weight and fat mass. This is directionally consistent with exercise-only evidence indicating that aerobic exercise has a clear dose–response association with reductions in body weight, waist circumference, and fat tissue (12). By contrast, resistance training may be particularly valuable when the priority is lean-mass preservation rather than maximal short-term fat loss. Taken together, the present findings support a modality-specific interpretation rather than a uniform assumption that TRE will potentiate every form of exercise to the same degree.

### 4.2 Effects of exercise combined with 16:8 time-restricted eating on glucose–lipid metabolism

This study found that exercise combined with a 16:8 TRE protocol significantly reduced triglycerides and LDL-C compared with exercise alone, but did not materially change total cholesterol, HDL-C, fasting glucose, fasting insulin, or insulin resistance. This pattern partially overlaps with prior syntheses. Dai et al. (30) reported favorable effects of combined TRE and exercise on metabolic health, whereas Sun et al. (14) found that TRE plus caloric restriction reduced body weight and fat mass more consistently than lipid markers. Thus, the present findings suggest that meal timing may add modest benefit to exercise for atherogenic lipids, but the effect is selective rather than comprehensive.

From a mechanistic standpoint, the 16:8 TRE protocol may modestly potentiate exercise-induced cardiometabolic adaptations by prolonging fasting-related lipolysis, reducing postprandial insulin exposure, and enhancing reliance on fatty-acid oxidation. Exercise simultaneously increases skeletal-muscle glucose uptake and lipid turnover, making an additive effect biologically plausible (33–35). However, the included trials were largely short, and many enrolled healthy rather than metabolically compromised adults, which may explain why glycemic indices changed little despite small improvements in triglycerides and LDL-C.

The subgroup finding that LDL-C reductions were more pronounced in men should be interpreted cautiously. The number of studies was limited, and sex differences may reflect baseline metabolic profile, exercise modality, or dietary adherence rather than biology alone. Likewise, the pooled SMDs for triglycerides and LDL-C were small. Therefore, combined TRE plus exercise should be viewed as a supportive lifestyle strategy rather than a substitute for established dyslipidemia treatment. Individuals might reasonably anticipate modest short-term improvements in fat loss and atherogenic lipid profiles, particularly when aerobic exercise is included and adherence is high.

Overall, these findings highlight the potential of combining exercise with 16:8 TRE as a pragmatic cardiometabolic strategy, while also underscoring the need for more precise prescription. Future studies should determine whether benefits differ by baseline metabolic risk, chronotype, eating-window placement, and exercise timing.

### 4.3 Practical implications

For generally healthy adults or adults with overweight/obesity already engaging in regular exercise, a 16:8 TRE schedule may be considered when the goal is modest short-term fat loss, provided that daily protein intake, training quality, and overall diet adequacy are maintained. Based on the current evidence, the most defensible expectation over 4–8 weeks is an additional reduction of approximately 1–1.5 kg in body weight and about 1 kg in fat mass beyond exercise alone, together with small improvements in triglycerides and LDL-C. These benefits are unlikely to be clinically transformative in isolation but may still be valuable as part of a broader cardiometabolic risk-reduction plan.

In clinical settings, intervention prescriptions should be individualized. Early-day eating windows may be preferable when feasible, yet the included studies did not allow formal testing of exercise timing or delivery setting. Likewise, too few studies reported whether interventions were supervised by exercise physiologists or research staff versus self-directed to permit a meaningful subgroup analysis. For individuals with dyslipidemia or obesity, combined TRE plus exercise may therefore be considered an adjunctive behavioral option rather than a replacement for standard clinical care.

Accordingly, any apparent subgroup differences, particularly those related to sex, should be interpreted with caution, as they may reflect underlying differences in baseline metabolic status rather than true sex-specific effects.

### 4.4 Study limitations

Several subgroup and meta-regression analyses were based on small numbers of studies, limiting statistical power. Most interventions lasted only 4–8 weeks, which is sufficient to detect short-term changes in body weight or fat mass but may be inadequate for more slowly changing outcomes such as glycemic control, insulin resistance, or sustained behavioral adaptation. In addition, improved exercise efficiency over time may attenuate further weight or fat loss even when cardiometabolic benefits persist, so the value of longer trials likely depends on the outcome under consideration. Reporting of exercise timing, eating-window timing, supervision level, and intervention delivery setting was often incomplete, precluding several clinically relevant moderator analyses. Finally, because the comparator was exercise alone rather than no treatment, the pooled estimates represent the incremental benefit of adding 16:8 TRE, not the total benefit of the combined intervention. Future trials should also examine whether TRE plus exercise can support weight-management maintenance alongside pharmacotherapy, particularly in the post-discontinuation phase when weight regain may follow cessation of GLP-1 receptor agonists (36).

Another important limitation relates to the restricted and unbalanced evidence base. Although 15 studies were included, most had relatively small sample sizes, and participant characteristics were not evenly distributed across sex and metabolic status. Specifically, male cohorts were predominantly composed of healthy individuals, whereas female cohorts were more often adults with overweight or obesity. This asymmetry limits the interpretability of subgroup comparisons and makes it difficult to disentangle potential sex-specific responses from differences in baseline metabolic condition. Therefore, the current evidence does not support broad generalization of the findings across both sexes or across underrepresented metabolic phenotypes, such as healthy women or men with obesity.

## 5 Conclusion

In summary, the currently available evidence suggests that combining exercise with a 16:8 TRE protocol may be associated with small short-term reductions in body weight, fat mass, triglycerides, and LDL-C compared with exercise alone. However, these findings are based on a limited number of studies with small sample sizes and an imbalanced distribution of participant characteristics across sex and metabolic status. Therefore, the results should be interpreted cautiously and should not be generalized across all adult populations. Larger and more diverse trials are needed to determine whether these effects are consistent across sexes and metabolic phenotypes.

## Data Availability

The original contributions presented in the study are included in the article/supplementary material, further inquiries can be directed to the corresponding author/s.
